# Q fever and coxiellosis in Brazil: an underestimated disease? A brief review

**DOI:** 10.1590/S1984-29612022051

**Published:** 2022-09-26

**Authors:** Eline Almeida Rodrigues de Souza, Marcos Rogério André, Marcelo Bahia Labruna, Mauricio Claudio Horta

**Affiliations:** 1 Laboratório de Doenças Parasitárias, Universidade Federal do Vale do São Francisco – UNIVASF, Petrolina, PE, Brasil; 2 Programa de Pós-graduação em Biociência Animal, Universidade Federal Rural de Pernambuco – UFRPE, Recife, PE, Brasil; 3 Laboratório de Imunoparasitologia, Departamento de Patologia, Reprodução e Saúde Única, Faculdade de Ciências Agrárias e Veterinárias – FCA, Universidade Estadual Paulista – UNESP, Jaboticabal, SP, Brasil; 4 Departamento de Medicina Veterinária Preventiva e Saúde Animal, Faculdade de Medicina Veterinária e Zootecnia – FMVZ, Universidade de São Paulo – USP, São Paulo, SP, Brasil

**Keywords:** Coxiella burnetii, zoonosis, infectious, small ruminants, humans, Coxiella burnetii, zoonose, infecciosa, pequenos ruminantes, humanos

## Abstract

Q fever, caused by the γ-proteobacterium *Coxiella burnetii,* is a zoonosis of great importance and global impact. This agent has high transmissibility and can spread over long distances via wind, in which a small number of aerosolized particles are needed to infect susceptible hosts. The clinical diagnosis of Q fever is difficult owing to the variety of clinical signs shared with other diseases. In Brazil, studies related to *C. burnetii* are constantly being conducted, and this review aims to increase the number of approaches already studied, leading to the following question: is Q fever an unknown, neglected disease, or does it have a focal occurrence in certain areas (exotic/rare) in the country?

## Resumo

A febre Q, causada pela γ-proteobactéria *Coxiella burnetii*, é uma zoonose de grande importância e impacto global. Este agente tem alta transmissibilidade e pode se espalhar por longas distâncias via vento, em que um pequeno número de partículas aerossolizadas são necessárias para infectar hospedeiros suscetíveis. O diagnóstico clínico da febre Q é difícil devido à variedade de sinais clínicos compartilhados com outras doenças. No Brasil, estudos relacionados à *C. burnetii* são constantemente realizados. Esta revisão visa aumentar o número de abordagens já estudadas, levando ao seguinte questionamento: a febre Q é uma doença desconhecida, negligenciada ou tem ocorrência focal em certas áreas (exóticas/raras) no país?

## Introduction

Q fever (QF), a disease with worldwide distribution, is among 13 zoonoses of global priority in terms of its impact on human health, responsiveness to interventions in livestock, clinical severity, and emergency, mainly in poor countries (Grace et al., 2012). QF is caused by *Coxiella burnetii,* a Gram-negative γ-proteobacterium belonging to the order Legionellales and the family Coxiellaceae, which multiplies exclusively intracellularly (Maurin & Raoult, 1999). In the last decade, the public health relevance of QF increased after an outbreak in 2007 in the Netherlands, where more than 4,000 people became ill (Schneeberger et al., 2014).

The most common route of transmission is through inhalation of contaminated aerosols (Guatteo et al., 2006). Humans who have direct contact with domestic ruminants or live close to rural locations are at a higher risk of contracting the infection as wind can contribute to the spread of the bacteria away from the primary infection area (Eldin et al., 2017; Miller et al., 2021a). Less often, the ingestion of unpasteurized milk or cheese can also be associated with infection by *C. burnetii* (Gale et al., 2015; Miller et al., 2021b), and transmission through tick bites among humans (Duron et al., 2015).

Considering that coxiellosis is an immediately notifiable disease in any animal species in Brazil (Brasil, 2013), the number of studies aimed at detecting infection by or exposure to *C. burnetii* in domestic and wild animals and contamination of milk and cheese has increased in the last few years. Nonetheless, the exact dynamics of the disease or the strain that circulates throughout the country are still unknown.

## Transmission

*Coxiella burnetii* is an obligate intracellular bacterium that presents resistance and environmental stability and is one of the most infectious microorganisms in humans (Eldin et al., 2017). Indeed, only 1–10 bacteria can infect and cause disease in a healthy person by the inhalation of contaminated aerosols (Centers for Disease Control and Prevention, 2019). Although this agent can replicate in various animal hosts, such as wild mammals, domestic mammals, birds, and arthropods (Maurin & Raoult, 1999), ruminants have been implicated as the main reservoirs for human infection (Pexara et al., 2018). When infected, these animals shed the infectious agent predominantly through vaginal secretions, milk, feces, urine, and semen (Guatteo et al., 2006). In addition, the pathogen can persist up to 1 month in refrigerated meat, in sheep wool for up to 10 months at 15-25 °C and up to 40 months at room temperature in powdered milk. Humans are primarily infected by contaminated aerosol inhalation (Gürtler et al., 2014).

Bacterium can infect placental trophoblastic cells and replicate at high densities in the placentas of ruminants and other mammals (Sánchez et al., 2006). A common clinical sign observed is abortion in sheep, goats, and women (Centers for Disease Control and Prevention, 2019). When mammals reach the end of gestation, healthy or unhealthy offspring are born. At this stage, *C. burnetii* spores sheds into the environment through the placenta and birth fluids. Therefore, *C. burnetii* can be transmitted directly during birth and contaminate the environment (Clark & Soares Magalhães, 2018).

Infection can reach up to 18 km from the point of origin via wind (Clark & Soares Magalhães, 2018). In addition, the number of people infected by an airborne pathogen depends on the force of emission, airborne transmission from the source to the receiver depending on weather and environmental conditions, and human exposure (duration, location, and physical activity). For instance, geographical areas with low vegetation and low soil moisture levels have higher levels of *C. burnetii* transmission (Van Leuken et al., 2016).

Other routes of transmission are rare, such as tick bites, ingestion of unpasteurized milk or dairy products, and person-to-person transmission (Centers for Disease Control and Prevention, 2019). However, there is still ongoing research about the importance of ingestion of contaminated milk and dairy products in the epidemiology of QF. Even though the infective dose needed for oral transmission of this agent is still unknown, a higher dosage is presumed to be necessary to allow a successful infection when compared to aerosol inhalation (Gale et al., 2015; Miller et al., 2021b). Epidemiological evidence of an association between QF outbreaks and the consumption of unpasteurized dairy products has been reported (Signs et al., 2012). In addition, since 1957, *C. burnetii* has been considered a chief microorganism to be eliminated from milk by exposure to high temperatures owing to its high heat resistance and pathogenicity (Enright et al., 1957).

## The role of ectoparasites in the transmission of Q fever

The presence of a hematophagous arthropod vector is not necessary for the transmission of *C. burnetii* but it is possible that this pathogen circulates between animals, with some participation of ectoparasites (Eldin et al., 2017). The role of ticks in the transmission of QF varies according to the epidemiological scenario. The importance of these arthropods is more significant in wildlife, as in rodents, birds, and lagomorphs, than in domesticated herds in developed countries (Eldin et al., 2017; The Center for Food Security and Public Health, 2017). The pathogen has been detected in several tick species, such as *Amblyomma* spp., *Rhipicephalus* spp., *Dermacentor* spp., *Ixodes* spp., *Hyalomma* spp., *Haemaphysalis* spp., in addition to other arthropods such as bed bugs, flies, and mites (Maurin & Raoult, 1999; Eldin et al., 2017). However, the common presence of *Coxiella*-like endosymbionts in ticks may cause overestimation of the prevalence of this agent (Duron et al., 2015).

When evaluated *in vitro*, tick infection by *C. burnetii* is typically systemic, as the bacteria have been detected in the midgut, hemolymph, Malpighian tubules, salivary glands, and ovaries (Lang, 1990). Indeed, these arthropods excrete *C. burnetii* in their body fluids and feces (up to 10^10^/g) (Philip, 1948). Thus, transmission of the agent by ticks to animals or humans can occur through dropping, direct contact, or bites (Duron et al., 2015).

*In vivo*, the ability of ticks to transmit *C. burnetii* depends on the tick population density, host preference and ecological constraints. Even if ticks are competent vectors in vitro, they can inefficiently transmit this pathogen *in vivo*. Moreover, they can act as key drivers of heterospecific transmission and the spatial spread of pathogens among vertebrates (Duron et al., 2015).

## Diagnosis and Genotyping

The virulence of *C. burnetii* is linked to lipopolysaccharide (LPS), found in two different phases on its surface. In phase I, LPS is in its complete virulence-bound form. Phase II represents the truncated and nonvirulent LPS form, resulting from phase I LPS after passages in cell culture, embryonated chicken eggs, or synthetic media in which it has lost its virulence (Moos & Hackstadt, 1987; Anderson et al., 2013). In epidemiological studies, it is preferable to search for anti-phase II antibodies that are always present during infection (Anderson et al., 2013). Some nonvirulent environmental strains express phase I LPS; additional factors are believed to contribute to virulence. Valvular disease and immunosuppression are known risk factors for QF endocarditis, with the understanding that host and environmental conditions also influence the outcome of the disease and clinical presentation of the infection (Honstettre et al., 2003).

In some cases, *Coxiella burnetii* can enter a latent phase and reappear as a more severe localized infection months or years later, as cases of chronic QF, regardless of the initial symptomatology or the severity of the disease during acute QF (Anderson et al., 2013; Eldin et al., 2017). It has been proposed that the term “persistent focused infection” should be adopted instead of “chronic QF” to identify the site where the infection is located (Eldin et al., 2017). Chronic QF is associated with phase I IgG antibodies (Wegdam-Blans et al., 2012).

According to the World Organisation for Animal Health - OIE (2018), there is no gold standard for the techniques used for diagnosis, although polymerase chain reaction (PCR) and enzyme-linked immunosorbent assay (ELISA) have been considered methods of choice. Comparing ELISA with indirect immunofluorescence assay (IFA), the latter is considered more sensitive and efficient in detecting IgM phase II antibodies, as ELISA is not as sensitive in detecting phase II antibodies, mainly when low antibody titers are found (Sahu et al., 2020). Commercial ELISA kits are available for the detection of phase I and II antibodies or phase-s pecific antibodies (Sahu et al., 2020). Although ELISA is less sensitive than IFA, owing to its ease of execution, it is widely used for screening a large number of samples (Miller et al., 2021a).

The use of molecular techniques for strain discrimination can aid in understanding the epidemiology of diseases and is a suitable tool for tracing outbreaks, such as multispacer sequence typing (MST) and multiple-locus variable-number tandem repeat analysis). Mioni et al. (2020b) observed, in Brazil, strains of *C. burnetii* with new genotypes by MST, namely CbCbB_F2 (detected in cattle fetuses), CbG_SVB22 (goat vaginal swabs), CbO_sn2 (sheep vaginal swabs), and Argentina (At12 - isolated from a tick), as well as genotypes already described in the literature. These findings suggest an independent evolution of the Argentinean strain and a common ancestor among Brazilian lineages, in addition to the two strains possibly introduced by the trade in animals and animal products.

Five types of plasmids have been described in *C. burnetii*: QpH1, QpRS, QpDV, QpDG, and an unnamed plasmid from a strain (CBQY) isolated in China. Pioneering studies have suggested that the plasmid content determines whether an isolate causes an acute or chronic disease (Dragan & Voth, 2020). In a virulence evaluation study of strains of the different origins inoculated into guinea pigs, five groups were evaluated; group I (QpH1 plasmid) strains expressing stage I LPS caused the most severe disease. The group II (QpH1 plasmid) strain, which is associated with acute human disease, appeared to be more virulent than the group I strains. The group III (QpH1 plasmid) strain appeared to be almost as virulent as the group I strain. Group IV (QpRS plasmid) strains were associated with persistent focused infections but appeared to be mildly virulent in the model studied. Group V (integrated plasmid sequence) strains are also associated with persistent and focused infections. Finally, group VI (QpDG plasmid) strains were shown to be avirulent owing to the absence of any clinical presentation of the disease, even producing phase I LPS, that are necessary for virulence. These findings suggest that *C. burnetii* may need additional virulence factors or may produce factors that attenuate virulence (Long et al., 2019).

## Risk factors associated with *Coxiella burnetii* infection

Monitoring *C. burnetii* infection in domestic ruminant herds is of great importance since these animals are the main reservoirs responsible for human outbreaks. Domestic ruminants can shed the bacteria in large quantities in birth products, urine, faeces and milk, without showing clinical signs (Eldin et al., 2017). Therefore, QF is often considered an occupational disease, as workers in direct contact with livestock (farmers, slaughterhouse workers, and veterinarians) have a higher risk of the disease (Miller et al., 2021a). Furthermore, people working in laboratories cultivating the bacterium, obstetricians who manage parturient women with QF, and the military also have higher risks (Eldin et al., 2017).

To reduce the risk, a set of measures on farms needs to be effective, such as vaccination (in Brazil there is still no availability of the vaccine), manure management, shearing management, segregated breeding area when sick animals are present, removal of risk material, prohibition of visitors, and control of other animal reservoirs (domestic and wild mammals) and ticks. Temporary changes in reproduction, identification, and slaughter of herds and the control of animal movements can influence transmission to humans (OIE, 2018). In the Northeast region, the rearing of domestic ruminants is carried out mainly by small producers, in a predominantly extensive and semi-extensive way, characterized by the use of native pasture and little increase in reproductive, sanitary and food management techniques (Alves et al., 2017). The animals are outside the premises part of the day and roam the wild environment to be able to feed, difficult to control the movement of animals.

## World Situation

QF is a reported disease worldwide, except in New Zealand, and several countries treat it as a nationally notifiable condition (Miller et al., 2021a). The prevalence is highly variable from one country to another owing to epidemiological disparities and/or the possibility of subnotification (Eldin et al., 2017). In areas of endemicity, QF occurs sporadically, usually after activities considered risky, such as agricultural activities and slaughterhouse works (Lautenschläger et al., 2000).

The first report of QF was in 1935, when an investigation of an outbreak of undiagnosed febrile illness among slaughterhouse workers was conducted in Brisbane, Queensland, Australia. First, the causative agent of the disease was thought to be a rickettsial agent called *Rickettsia burnetii*. While wild animals were considered the main reservoirs, domestic animals were implicated as secondary reservoirs, and the disease was assumed to be transmitted by ticks or other arthropods. After many studies, it was concluded that the causative agent was a bacterium belonging to the order Legionellales and was renamed *C. burnetii* (Maurin and Raoult, 1999). In Australia, 400–600 cases per year were reported between 2003 and 2017 (Tozer et al., 2020).

In 1940, 15 individuals were infected in the United States after the initiation of QF studies at the National Institutes of Health. Later, in 1946, 47 individuals in the same locality developed the disease. It is believed that the reason for these cases was the incorrect handling of the bacteria, resulting in the release of the agent into the air of the facility, as not all infected people worked directly with the disease (Hirschmann, 2019). Starting in 1999, cases in the country were reported to the Centers for Disease Control and Prevention, with the number of cases varying between 164 and 215 in 2016 and 2018, respectively (Miller et al., 2021a).

Africa was the third continent to document QF in 1955, where nine countries reported varying numbers of infected individuals (Eldin et al, 2017). In the same year, the disease spread to South America, with the first case documented in a slaughterhouse in Cayenne, French Guiana. Sporadic cases have occurred in South America over the years, with 150 cases per 100,000 inhabitants in 2005 (Eldin et al., 2014).

Between 2007 and 2010, the largest QF epidemic ever occurred in the Netherlands. Seasonal outbreaks have resulted in at least 4,000 known acute cases and an estimated total of 40,000 cases (Delsing et al., 2010; Dijkstra et al., 2012). Not all infected people worked directly with animals but were located downwind on dairy farms (Kampschreur et al., 2014; Oliveira et al., 2017). Although a public health strategy was implemented, it was not successful, and it was decided to systematically slaughter pregnant goats and sheep, with more than 50,000 animals being slaughtered (Schneeberger et al., 2014). To control the disease, vaccination of animals was implemented (Roest et al., 2011).

## Situation in Brazil

**Figure 1:**
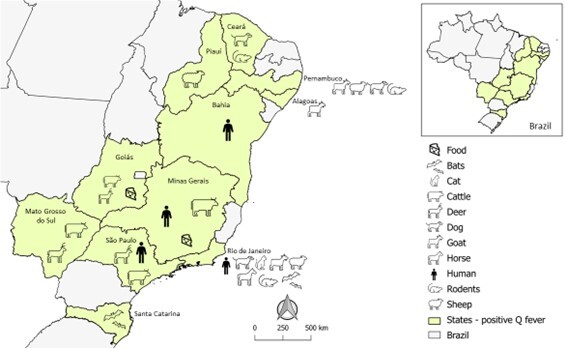
Serological and/or molecular detection studies of *Coxiella burnetii* carried out in Brazil.

Studies in Brazil have been conducted on animals (domestic and wild), humans, and food (Table 1 and Figure 1), as well as the genotypic evaluation of bacteria. They began in the 1950s, when Brandão et al. (1953) investigated meat handlers and, as a control, employees of a glass factory in São Paulo state. The authors found antibodies in 1.56% of the total samples analyzed (meat handlers and glass factory workers), with endpoint titers ranging from 4 to 8 in the complement fixation test (no phase I or phase II specification), concluding that *C. burnetii* was circulating at this location. Then, 71 cattle handlers were examined for the presence of anti-*C. burnetii* antibodies, which were found in five (7%) of these patients through the complement fixation test, with endpoint titers ranging between 8 and 16, no phase I or phase II specification (Valle et al., 1955).

**Table 1:** Serological and/or molecular detection studies of *Coxiella burnetii* carried out in Brazil.

Year	Authors	Diagnosis	Animal	State or region
*Humans*				
1953	Brandão et al.	CFT	-	São Paulo
1955	Valle et al.	CFT	-	São Paulo
1974	Riemann et al.	MA	-	Minas Gerais
2005	Costa et al.	IFA	-	Minas Gerais
2006	Costa et al.	IFA	-	Minas Gerais
2008	Siciliano et al.	IFA	-	Bahia
2009	Lamas et al.	IFA	-	Rio de Janeiro
2011	Lemos et al.	IFA/PCR	-	Rio de Janeiro
2012	Rozental et al.	IFA/PCR	-	Rio de Janeiro
2013	Lamas et al.	PCR	-	Rio de Janeiro
2015	Siciliano et al.	IFA/IHQ	-	São Paulo
2016	Mare-Guia et al.	PCR	-	Rio de Janeiro
2018	Lemos et al.	IFA/PCR	-	Rio de Janeiro
2018a	Rozental et al.	IFA	-	Rio de Janeiro
2022	Meurer et al.	IFA/PCR	-	Minas Gerais
*Domestic animals*				
1955	Valle et al.	CFT	Cattle	São Paulo
2014	Mares-Guia et al.	IFA/PCR	Dog, cat, goat, sheep and horse	Rio de Janeiro
2017	Guimarães et al.	IFA	Sheep	Piauí
2018	Oliveira et al.	ELISA/PCR	Goat	Alagoas
2018	Souza et al.	IFA	Goat and sheep	Pernambuco
2019a	Zanatto et al.	IFA	Cattle	Goiás, São Paulo, Minas Gerais, Mato Grosso do Sul
2020a	Mioni et al.	IFA/PCR	Cattle	São Paulo
2020	Ramos et al.	IFA	Cattle	Mato Grosso do Sul
2020	Oliveira et al.	IFA	Dog	Ceará, Pernambuco
2022	Mioni et al.	PCR	Cattle	São Paulo
*Wild animals*				
2017	Rozental et al.	PCR	Rodents	Rio de Janeiro
2018	Ferreira et al.	PCR	Bats	Rio de Janeiro, Santa Catarina
2019b	Zanatto et al.	IFA/PCR	Deer	Mato Grosso do Sul, São Paulo
2020	Oliveira et al.	IFA	Rodents	Ceará, Pernambuco
*Food*				
2018b	Rozental et al.	PCR	-	Minas Gerais
2019	Mioni et al.	PCR	-	Goiás
2020	Rozental et al.	PCR	-	Minas Gerais
2021	Nascimento et al.	PCR	-	Minas Gerais

CFT: Complement Fixation Test; ELISA: Enzyme Immunoabsorption Assay; IFA: Indirect Immunofluorescence Assay; IHQ: immunohistochemical; MA: Microagglutination; PCR: Polymerase Chain Reaction.

Since then, other studies have been developed, even with a large space of time between them. After 19 years in Minas Gerais State, the presence of anti-*C. burnetii* antibodies in 22% of 219 students was reported at the Belo Horizonte School of Veterinary Medicine, with endpoint titers ranging from 4 to 64 using the microagglutination test, no phase I or phase II specification (Riemann et al., 1974). In 2005, the studies became more consolidated in the country, and a study including 437 people from the municipality of Piau, Minas Gerais reported the presence of anti-*C. burnetii* antibodies in 3.9% of participants by the IFA test, using phase I and phase II antigens, with titers ranging from 64 to 128. Of these, there were multiple/cross-reactive samples between *C. burnetii* and *Bartonella henselae* (11.8%), *Bartonella quintana* (5.9%), *Rickettsia rickettsii* (11.8%), *Rickettsia typhi* (11.8%), and *Ehrlichia chaffeensis* (11.8%). The local economy of that municipality was linked to livestock; the entire population has continuous contact with several domestic animals, and tick attack history is widespread, affecting approximately 100% of the population (Costa et al., 2005).

These epidemiological studies were initiated with humans who were at risk of development of the disease, that is, those who had direct contact or were close to animals, mainly cattle, goats, and sheep, which are the main hosts of the bacterium (Maurin & Raoult, 1999). Accordingly, other studies have been developed on the same topic with the objective of understanding QF in some regions. Another high-risk group is immunosuppressed individuals. In Rio de Janeiro, the anti-*C. burnetii* antibodies by IFA test, no phase I or phase II specification, was observed in 3.2% of 125 HIV-positive persons, with titer endpoints between 64 and 128 (Lamas et al., 2009), and 9.3% of 300 people who injected drugs. These findings suggest that the exposure to *C. burnetii* may be related to non-hygienic injection practices, immunosuppression, and sharing of injection paraphernalia (Rozental et al., 2018a).

In 2008, the first case report in Bahia was published, in which a patient with a history of dyspnea worked in cattle fields and frequently consumed raw milk and its derivatives. Consequently, it was confirmed through IFA the presence of anti-*C. burnetii* antibodies with titers above 1,600 (phase I). Despite the recommended treatment with doxycycline and ciprofloxacin due to the possibility of *C. burnetii* endocarditis, 3 weeks later the symptoms returned, and the patient subsequently died (Siciliano et al., 2008). Two years later, in Rio de Janeiro, a case of QF was confirmed by conventional PCR (based on *htpAB* gene) and two samples, collected at different times (40 and 70 days after onset of the illness), were seropositive by the IFA test (endpoint titers to phase II antigen of 256 and 1,024, respectively) in a man with a 40-day fever associated with thrombocytosis. Because it is an easily transmitted disease, all residents of the households, including animals, were tested, and anti-*C. burnetii* antibodies were detected in his wife (titer of anti-phase II IgG of 128) and two dogs (titers of anti-phase II IgG of 64 and 128) (Lemos et al., 2011).

Other studies have been related to the symptoms found in some patients, as QF has non-specific symptoms and can be confused with other diseases. In this study, 726 patients with a history of fever treated at five health centers in Juiz de Fora, Minas Gerais State, were evaluated. Of these, 2.2% were seroconverted to *C. burnetii,* with titers ranging from 32 to 2048, phase I and phase II (Costa et al., 2006). In 2018, an assessment was published in Rio de Janeiro on five cadets with febrile illness after the military survival training camp, where it was reported that they had numerous tick bites, and 45 cadets directly participated in the slaughter of animals for food in the field, including goats. Five cadets were seroconverted to anti-*C. burnetii* antibodies and reached an endpoint titer of 16,384 (phase II). In addition, *C. burnetii* DNA was detected by PCR in a bronchoalveolar lavage sample from one cadet (Lemos et al., 2018).

Therefore, in 2012 in Rio de Janeiro, a man diagnosed with severe pneumonia presented with anti-*C. burnetii* antibodies with a titer endpoint of 512 in the IFA test (phase I and phase II). Additionally, *C. burnetii* DNA was detected in bronchoalveolar lavage using the conventional PCR-based *IS1111* gene, which is the first case of DNA detection in this type of sample (Rozental et al., 2012). In 2013, 51 heart valve samples from surgical patients were collected in Rio de Janeiro, of which one sample was positive for *C. burnetii* by conventional PCR (Lamas et al., 2013). Two years later, in São Paulo, an investigation was published of 221 patients with endocarditis, 1.8% of whom were seropositive with an endpoint titer of 800 (phase I) by the IFA test. In addition, 51 of these 221 patients were culture-negative for *C. burnetii* and were evaluated by immunohistochemistry of the heart valves for *C. burnetii*, resulting in 7.8% positivity (Siciliano et al., 2015).

In 2016, the presence of *C. burnetii* was investigated after an outbreak of dengue in Rio de Janeiro, where the first molecularly identified Brazilian case of QF was reported in 2008. Conventional PCR (using primers targeting the IS1111 transposase elements) was performed on the blood of 272 patients with clinical suspicion of dengue, in which *C. burnetii* DNA was found in 3.3% of these patients. The authors concluded that despite most samples being negative in the molecular test, the diagnosis of QF cannot be excluded, and that the number of infected individuals is probably much higher than the finding because only the molecular analysis of the blood was performed and there was not enough sample for serological analysis (Mares-Guia et al., 2016). In 2021, in Minas Gerais, 437 patients suspected and later confirmed to be negative for dengue were evaluated, 4.8% of whom were seropositive for anti-*C. burnetii* antibodies on the IFA test, with titers ranging between 64 and 128 (phase I and phase II). This study showed that rural residence was a risk factor for QF in that region (Meurer et al., 2022).

In domestic animals, research is more related to the epidemiology of the disease in the regions studied and its possible impact on public health. These studies began in 1955 in São Paulo, when Valle et al. (1955), in addition to evaluating cattle handlers, also tested 171 cattle, obtaining a result of 14% of these animals being positive for anti-*C. burnetii* antibodies, with endpoint titers varying between 16 and 256 (no phase I or phase II specification). After 34 years, a study was conducted with 76 goats from five states in the northeast region, all of which were negative (Brown et al., 1989).

In Rio de Janeiro, a study was carried out with 14 dogs, 1 cat, 10 goats, 3 sheep and 2 horses, in addition to bodily secretions (milk, vaginal swab, and anal swab) from goats. Of these, the anti-*C. burnetii* antibodies was observed through the IFA test (phase I and phase II) in two (14.3%) dogs with endpoint titers of 64, five (50%) goats with endpoint titers ranging from 64 to 128, and two (66.7%) sheep with endpoint titers of 64, as well as bacterial DNA in six (60%) milk samples, two (14.3%) dog blood samples, and two (20%) goat blood samples (Mares-Guia et al., 2014). In 2017, the presence of 1.96% of anti-*C. burnetii* antibodies using the IFA test (no phase I or phase II specification) from 153 sheep studied in Piauí State with titers ranging from 64 to 4,096 was confirmed (Guimarães et al., 2017). In 2018, seropositivity of 55.12% for *C. burnetii* among 312 goats was reported, in Alagoas State, through the ELISA test, with 65.7% of the serorreactives presenting high titers (≥ELISA++). Furthermore, bacterial DNA was detected by nested PCR (using specific primers to amplify the IS1111 gene) in 8.7% of 23 goat placenta samples, which were also seroreactive (Oliveira et al., 2018), and 2.2% and 2.1% of anti-*C. burnetii* antibodies, by IFA test (no phase I or phase II specification), from 412 goats and 403 sheep from Pernambuco, respectively, with endpoint titers ranging from 64 to 65,536 (Souza et al., 2018). In 2019, in the evaluation of *C. burnetii* associated with bovine viral diarrhea virus (BVDV), bovine herpesvirus (BoHV), *Leptospira* spp., *Neospora caninum*, *Toxoplasma gondii* and *Trypanosoma vivax* in reproductive disorders in cattle originating from São Paulo, Minas Gerais, Mato Grosso do Sul, and Goiás, 13.7% of 102 cattle evaluated presented anti-*C. burnetii* antibodies, with high titers reaching 131,072 (phase I). Among these seropositive animals, 10% were from Goiás (n=20), 12.5% from São Paulo (n=32), 17.5% from Minas Gerais (n=40), and 10% from Mato Grosso do Sul (n=10) (Zanatto et al., 2019a).

In the Northeast region, a low rate of seropositivity to *C. burnetii* was found in the Sertão region (Souza et al., 2018), in contrast to the high rate of seropositivity in the Agreste region (Oliveira et al., 2018). Both have a semi-arid climate; however, Sertão has higher temperatures and lower humidity than Agreste (IBGE, 2012; Moura et al., 2007). Whether the temperature influenced these cases remains to be investigated since hot and dry climatic conditions could facilitate dispersion of the bacteria (Nusinovici et al., 2015). In the Southeast region, cattle from 54 cities in the state of São Paulo sent to the slaughterhouse were evaluated in 2020. Of these 54 cities, 83.3% had at least one seropositive animal and 23.8% from 1,515 samples collected. Furthermore, seropositive samples were subjected to real-time PCR based on the IS1111 gene, and 12.2% of serum samples were positive (Mioni et al., 2020a). Furthermore, in 2020, anti-*C. burnetii* antibodies were detected by IFA in 1% of 200 beef cattle in a study related to co-seropositivity among tick-transmitted agents in five different farms located in the central region of the Pantanal Sul-Matogrossense (Ramos et al., 2020). Seroreactivity to *C. burnetii* was observed in 5% (endpoint titers 128) and 4% (titers 256–512) of domestic dogs from Pernambuco and Ceará, respectively, using the IFA test, no phase I or phase II specification (Oliveira et al., 2020). In 2022, a tissue evaluation study of aborted fetuses and possible co-infections was published, in which the DNA of *C. burnetii* was detected by real-time PCR targeting the IS1111 gene in 9.2% of 76 fetuses studied in São Paulo (Mioni et al., 2022).

Studies in wild animals began in 2017 when 131 rodents belonging to 18 species were evaluated in Rio de Janeiro, with 4.6% positivity by conventional PCR targeting the IS1111 gene in four species of rodents (*Akodon cursor, Mus musculus, Oxymyxterus dasytrichus* and *Oligoryzomys nigripes*) (Rozental et al., 2017). Subsequently, Ferreira et al. (2018) evaluated 119 bats belonging to 21 species from the states of Rio de Janeiro, Bahia, and Santa Catarina, and found *C. burnetii* DNA in 3.4% by conventional PCR targeting the IS1111 gene in *Artibeus lituratus* and *Artibeus fimbriatus*, in Rio de Janeiro and Santa Catarina. In addition, in 2019, a study was conducted on free-living deer in the states of Mato Grosso do Sul, Goiás, São Paulo, and Paraná in which anti-*C. burnetii* antibodies were found in 5.32% of 169 animals by the IFA test (phase I), with titers ranging from 256 to 16,384, in the regions of Mato Grosso do Sul and São Paulo. The seropositive species were *Mazama gouazoubira* and *Blastocerus dichotomus* (Zanatto et al., 2019b). In 2020, in the regions of Pernambuco and Ceará, one rodent (*Wiedomys pyrrhorhinos*) and one marsupial (*Didelphis albiventris*) were seroreactive to *C. burnetii* at endpoint titers of 128 and 4,096, respectively, by IFA test, no phase I or phase II specification (Oliveira et al., 2020). Finally, in 2021, Mato Grosso do Sul, Ikeda et al. (2021) sampled 135 non-hematophagous bats and did not find real-time PCR positivity for *C. burnetii* based on the IS1111 gene. Therefore, bats do not act as important hosts in epidemiology or *C. burnetii* in Brazil. Recently, *C. burnetii* DNA was not detected in blood and spleen samples from 397 free-living Xenarthra mammals (233 sloths, 107 anteaters, and 57 armadillos) in five Brazilian states (Mato Grosso do Sul, São Paulo, Pará, Rondônia, and Rio Grande do Sul) using a qPCR assay based on the IS1111 gene (Oliveira et al., 2022).

Studies focusing on food were only published in 2018, when *C. burnetii* DNA was detected in 9.43% of 53 artisanal cheese samples from the agroindustries of the Serro microregion, Minas Gerais, by nested PCR to amplify the IS1111 gene (Rozental et al., 2018b; 2020). Subsequently, Mioni et al. (2019) evaluated 112 bulk cow milk samples from Goiás and found *C. burnetii* DNA in 3.6% of these samples using real-time PCR targeting the IS1111 gene. Finally, Nascimento et al. (2021) found the bacterial DNA in 4.6% of 87 samples of artisanal cheese in Minas Gerais using real-time PCR based on the IS1111 gene.

For being an easily transmissible bacterium, what draws attention in most of the epidemiological investigation studies of *C. burnetii* is the low serological percentage in herds (usually only one or two seropositive animals), as herds represent animals that are always reared together in a farm of goat and sheep (Souza et al., 2018). Why would this happen in a situation in which the spread of the bacteria could be facilitated? Another study in the Pernambuco region, where risk factors for the disease were investigated, reported more seropositive goats and sheep but with a low percentage rate (unpublished data for Souza et al.). Anti-*C. burnetii* antibodies can be detected in the sorologic analysis between 2 and 3 weeks after exposure (Roest et al., 2013), and the presence of a high titer is suggestive of recent exposure. But some animals do not appear seroconverted, and others shed organisms before developing antibodies (The Center for Food Security and Public Health, 2017). Oliveira et al. (2018) found seropositivity above 50% using a commercial ELISA test to detect the presence of anti-*C. burnetii* phase I and II antigen IgG antibodies in goats, differentiating them from others that use IFA or complement fixation (CF). In addition, Mares-Guia et al. (2014) found a relatively high rate of 50% in goats and 66.6% in sheep using the IFA test; however, few animals were evaluated (10 and 3, respectively). In a comparative study of the IFA, ELISA, in goats, the authors concluded that the IFA test had a high sensitivity and specificity and should be used as a reference diagnostic test for the detection of antibodies against *C. burnetii* in goats and other animals (Muleme et al., 2016). However, ELISA has a greater and faster facility for testing large numbers of samples (Muleme et al., 2016).

Bacterial DNA can be detected in the blood by PCR within 2 weeks of infection (Wegdam-Blans et al., 2012). Therefore, caution should be exercised with the interpretation of results obtained through this technique, after which the sensitivity can rapidly decrease and lead to false negatives because they are below the detection limit (Miller et al., 2021a). Infected animals shed the infectious agent predominantly through feces, urine, saliva, vaginal discharge, the placenta, and amniotic fluid (Abiri et al., 2019). However, reproductive secretions are more important, being the main choice for detection of the pathogen DNA and play a more prominent role in the transmission and spread of bacteria (Berri et al., 2000). It has been observed that there is greater sensitivity in the detection of *C. burnetii* in vaginal swab samples, whereas specificity is greater in milk samples from goats and sheep (Abiri et al., 2019). Most studies have chosen the insertion sequence of the IS1111 gene as the target for detection of *C. burnetii* by PCR because it is a repeating element of multiple copies with 7–110 copies per isolate, allowing a greater sensitivity of the technique (Sahu et al., 2020). Studies have recommended that serological testing in combination with PCR of samples most suitable for the detection of *C. burnetii* is indispensable for the definitive diagnosis of acute QF and for estimating the true rate of infection at the herd or population level (Schneeberger et al., 2010).

One hypothesis for the generally low positivity for *C. burnetii* in Brazil is the low virulence of local strains; therefore, systematic typing can be performed to aid the identification and monitoring of the virulent strains (Mioni et al., 2020b). It was observed that there are new strains of *C. burnetii* circulating in some parts of the country, which may have been introduced by trade in animals and animal products. Do the strains identified by Mioni et al. (2020b) match those detected in the northern hemisphere? This highlights the importance of evaluation of the virulence of these strains of different lineages in QF cases to understand how the disease occurs in the country. This study elucidates the role of genomic content and virulence of the bacterium (Mioni et al., 2020b).

## Final Remarks

In this study, we showed that there is a circulation of *C. burnetii* in Brazil. However, further studies are needed to understand the dynamics of this disease in the country.

In this sense, what would justify the finding of a few infected animals in the herd since the main transmission is through the aerogenic route? Are ambient temperature and humidity directly related to agent maintenance and transmission? Owing to the severity of the disease, should competent institutions make it compulsorily notifiable in humans?

In addition, serological and molecular tests (in samples that are more likely to lead to detection of the pathogen even in asymptomatic animals, such as vaginal swabs and milk samples) should be employed in conjunction, as the presence of the bacterium may be being underestimated, and thus observe a reliable diagnosis, especially in epidemiological studies. Also, a genotyping test could be applied to obtain better knowledge of the strains at different locations where QF was studied.

Differential diagnosis of reproductive disorders is recommended in bovine herds, which can occur by BVDV, BoHV, *Leptospira* spp., *N. caninum*, *T. gondii* and *T. vivax* (Zanatto et al., 2019b), and in goat and sheep herds, which include infectious agents such as *T. gondii*, *Chlamydophila abortus*, *Brucella* spp., *Listeria monocytogenes*, *Campylobacter* spp., *Salmonella* spp., *Leptospira* spp. and *N. caninum* (Pereira et al., 2013; Gazzonis et al., 2016).

Although in some regions of the country, the expected frequency related to infection for *C. burnetii*, a pathogen of high importance and transmissibility, was not found, it does not reduce the risk of occurrence of the disease, and it is necessary to include the diagnosis in routine practice to promote preventive measures and adequate treatment.
